# NEW MEASURES OF MULTIMORBIDITY BASED ON A THEORY OF DEPENDENT COMPETING RISKS

**DOI:** 10.1093/geroni/igae098.2812

**Published:** 2024-12-31

**Authors:** Igor Akushevich, Arseniy Yashkin, Julia Kravchenko, Anatoliy Yashin

**Affiliations:** Duke University, Durham, North Carolina, United States; Duke University, Durham, North Carolina, United States; Duke University School of Medicine, Durham, North Carolina, United States; Duke University, Durham, North Carolina, United States

## Abstract

The phenomenon of multimorbidity is increasingly common among older U.S. adults, however the driving forces behind multimorbidity development remain unclear. In this study we develop a novel approach for the identification and explanation of causal pathways generating multimorbidity patterns in the population of older U.S. adults. Our approach is based on the evaluation of the dependent competing risks among diseases potentially determining the process of generating a given multimorbidity cluster using parametric and non-parametric methods. This approach allows for modeling the joint distribution of time-to-disease-onsets with mutual correlations; characterizing cluster properties such as speed of cluster formation and their prevalence in the population. This joint distribution is estimated using individual morbidity trajectories extracted from Medicare administrative claims data and dynamically constructed for all ages resulting in an age-specific-function of multimorbidity generation. The identified clusters and estimated properties were compared to those obtained using traditional methods (e.g., cluster analysis or latent structure analyses). Our method strongly outperforms the current methods of identification of multimorbidity clusters which were characterized by approximate cluster composition with no room for improvement without additional methodologic assumptions. The substantive results of this study encompass the identification of multimorbidity clusters and their properties and allows extensions for the quantification of the effects of risk factors on these clusters (including evaluating causality), the provision of causal explanations for disparities in multimorbidity, and the development and testing of novel approaches for multimorbidity analysis through simulation studies.

